# A Fully Human Derived Monoclonal Antibody Provides Potent Pre‐ and Postexposure Protection Against Rabies Virus

**DOI:** 10.1155/tbed/3021186

**Published:** 2026-06-19

**Authors:** Ruo Mo, Jingqi Xu, Huanqin Zheng, Tianyi Yin, Haiyang Cong, Lugong Chen, Feihu Yan, Na Feng, Tiecheng Wang, Jianzhong Wang, Yongkun Zhao, Xianzhu Xia

**Affiliations:** ^1^ College of Veterinary Medicine, Jilin Agricultural University, Changchun, 130118, Jilin, China, jlau.edu.cn; ^2^ Key Laboratory of Jilin Province for Zoonoses Prevention and Control, State Key Laboratory of Pathogen and Biosecurity, Academy of Military Medical Sciences, Changchun, Jilin Province, China, bmi.ac.cn

**Keywords:** monoclonal antibody, phage display, rabies

## Abstract

Rabies remains a critical global health concern, particularly in endemic regions where timely access to postexposure prophylaxis (PEP) is often limited. The effectiveness of PEP relies heavily on rabies immune globulin (RIG), yet plasma‐derived products continue to face persistent issues of limited supply, variable potency, and high cost. These constraints have intensified the demand for recombinant monoclonal antibodies (mAbs) that provide consistent quality and scalable production. Here, we describe the development of a fully human mAb, H81L90, directed against the rabies virus glycoprotein (RABV‐G). The antibody was isolated from a phage‐display library constructed using peripheral blood mononuclear cells (PBMCs) from vaccinated donors. H81L90 exhibited strong, specific binding to native RABV‐G with nanomolar affinity as determined by surface plasmon resonance (SPR) analysis. In cell‐based neutralization assays, H81L90 efficiently blocked infection by the ERA‐enhanced green fluorescent protein (EGFP) strain, achieving complete viral inhibition at low microgram concentrations. Protective efficacy was subsequently evaluated in a murine challenge model, where a single intramuscular injection of H81L90 conferred full survival when administered before or at the time of viral exposure and retained measurable activity at reduced doses postexposure. Histopathological assessment revealed substantially lower viral antigen in hippocampal tissue from treated animals, indicating suppression of early neuroinvasion. Collectively, these data establish H81L90 as a potent, fully human antibody with both preventive and postexposure prophylactic potential, supporting its continued development as a next‐generation biologic to complement or replace current RIG formulations in rabies PEP.

## 1. Introduction

Rabies continues to impose a substantial global health burden, claiming an estimated 59,000 lives each year, primarily in Asia and Africa, where dog‐mediated transmission persists as the main route of infection [1, 2]. Once clinical symptoms appear, rabies is almost invariably fatal, which makes timely postexposure prophylaxis (PEP) the only effective means of preventing disease onset [3]. Despite international initiatives, including the World Health Organization’s goal to eliminate dog‐mediated human rabies by 2030, many endemic regions still face persistent challenges, such as limited vaccine accessibility, insufficient surveillance, and particularly the scarcity of rabies immune globulin (RIG) [2, 4]. These obstacles continue to undermine the success of PEP programs in regions where the disease remains entrenched.

The rabies virus (RABV), a member of the *Lyssavirus* genus, primarily enters through bite wounds, replicates locally, and exhibits strong neurotropism, traveling retrogradely along nerves to the central nervous system, where it causes fatal encephalitis, which possesses a single‐stranded, negative‐sense RNA genome that encodes five structural proteins. Among them, the surface glycoprotein (RABV‐G) plays a pivotal role in host receptor recognition and viral fusion, and it is the exclusive target for virus‐neutralizing antibodies [5–8]. Multiple antigenic sites (sites I–IV, along with G1 and G5 regions) have been identified on RABV‐G, with sites I and III representing major neutralizing epitopes [9, 10]. Residue 333 within site III is of particular biological interest as substitutions at this position have been linked to changes in host range and neurovirulence [11]. Recent structural and cryo‐electron microscopy studies have revealed that many potent neutralizing antibodies recognize the prefusion conformation of RABV‐G, reinforcing its role as a critical immunogen and an ideal target for rational antibody design [8, 9].

RIG remains an essential component of PEP for category III exposures. However, plasma‐derived human (HRIG) and equine RIG (ERIG) preparations are constrained by high production costs, limited global availability, and the potential for adverse reactions [12, 13]. Furthermore, HRIG manufacturing depends on human plasma donations, while ERIG production involves repeated animal immunizations, both of which restrict scalability and raise ethical considerations. The development of recombinant monoclonal antibodies (mAbs) offers a promising path forward. mAbs provide uniform potency, consistent quality and can be produced in large quantities through cell‐based expression systems, overcoming many of the logistical and safety limitations associated with conventional RIG.

In recent years, multiple antibody‐based strategies against RABV have progressed toward clinical evaluation, highlighting the feasibility of replacing traditional RIG in PEP. These approaches include single mAbs, such as Rabishield (17C7) and Ormutivimab (NM57), antibody combinations designed to minimize viral escape, such as Twinrab, SYN023, and CL184, and next‐generation engineered formats, including broadly neutralizing antibodies (e.g., RVC20 and RVC58) and bispecific antibodies that simultaneously target distinct glycoprotein epitopes (e.g., GR1801). Collectively, these advances illustrate the ongoing transition from polyclonal immunoglobulin preparations to rationally designed monoclonal and multispecific antibody therapeutics for rabies PEP [14–25]. The World Health Organization recommends that antibody combinations target nonoverlapping epitopes to prevent viral escape and ensure broad cross‐lineage protection [8].

In this study, we constructed a phage display antibody library using peripheral blood mononuclear cells (PBMCs) from rabies‐vaccinated donors and identified a fully human mAb, H81L90, with high affinity and potent neutralizing activity against RABV. We characterized its antigen‐binding properties, neutralization potential, and protective efficacy in a murine model. The results support H81L90 as a promising next‐generation biologic capable of complementing or replacing RIG in rabies PEP, particularly in low‐resource settings where access to plasma‐derived immunoglobulins remains severely limited.

## 2. Materials and Methods

### 2.1. Cell Lines and RABV Strains

Baby hamster kidney cells (BHK‐21 and the BSR‐T7/5 derivative cell sourced from Changchun Veterinary Research Institute under a license) lines were used for RABV propagation and neutralization assays. Cells were maintained in DMEM (Fisher, 11574486) supplemented with 10% fetal bovine serum (FBS), 10 U/mL penicillin, and 10 µg/mL streptomycin. HEK293T and 293F cells were cultured in DMEM (Gibco) with 10% FBS and 1% penicillin–streptomycin under standard incubation conditions (37°C, 5% CO_2_). Routine mycoplasma screening was performed to ensure cell‐line authenticity and contamination‐free status. A recombinant ERA‐GFP strain, generated through a reverse genetics system and expressing enhanced green fluorescent protein (EGFP) [26], was used for live virus neutralization assays. Viral titers were determined in BHK‐21 cells by direct fluorescent antibody assay (DFA) and expressed as TCID_50_/mL. The fixed RABV strain CVS‐11 (GenBank EU126641.1) was propagated in BHK‐21 cells and stored at −80°C at the Changchun Veterinary Research Institute, Chinese Academy of Agricultural Sciences. Human peripheral blood samples used for subsequent PBMC isolation were collected with informed consent in accordance with institutional ethical guidelines. Recombinant RABV‐G was expressed and purified using a baculovirus expression system (sourced from the Changchun Veterinary Research Institute under license).

### 2.2. Isolation of PBMCs From Rabies Vaccinated Individuals

Healthy adult volunteers who had received a complete course of human diploid cell rabies vaccine were recruited under an approved protocol, and written informed consent was obtained prior to blood collection. Individuals vaccinated within the previous year were given a single booster dose, while those without recent immunization received the full three‐dose regimen. Virus‐neutralizing antibody titers were evaluated 1 week after each vaccination to confirm an adequate immune response. Approximately 100 mL of peripheral blood was collected from each donor 1 week after the final immunization. PBMCs were separated by Ficoll‐Paque density gradient centrifugation, washed twice with phosphate‐buffered saline (PBS), and resuspended at a final density of 5 × 10^5^ cells/mL. The purified PBMCs were preserved in the TRIzol reagent for RNA stabilization and stored in liquid nitrogen until further use. These PBMC preparations were subsequently utilized for phage display library construction and screening of antibody fragments specific for the RABV glycoprotein (RABV‐G).

### 2.3. Construction and Biopanning of Phage Display Antibody Library

A human phage display antibody library was established using PBMCs isolated from rabies vaccine‐immunized donors. Total RNA was extracted using the TRIzol reagent and immediately reverse transcribed into complementary DNA (cDNA), which served as the template for amplification of variable heavy (VH) and variable light (VL) chain regions. PCR reactions were carried out using degenerate primer sets covering all known human immunoglobulin germline families. The amplified VH and VL fragments were linked via a (Gly_4_Ser)_3_ flexible peptide linker to form single‐chain variable fragments (scFvs). The assembled scFv sequences were ligated into the pCOM3XSS phagemid vector in‐frame with M13 gene III for display on the phage surface. Recombinant plasmids were transformed into *E. coli* TG1 cells by electroporation, and the transformants were infected with helper phage VCSM13 to rescue the scFv‐displaying phage particles.

For the selection of RABV‐G–specific binders, purified RABV‐G was immobilized on immunotubes and incubated with the phage library at room temperature. After extensive washing with PBS containing 0.05%–0.1% Tween‐20 to remove weakly bound or nonspecific phages, the remaining bound particles were eluted using 0.1 M glycine–HCl buffer (pH 2.2) and immediately neutralized with 1 M Tris‐HCl (pH 8.8). The eluted phages were amplified in fresh *E. coli* TG1 cultures and subjected to additional rounds of biopanning with progressively increased washing stringency to enrich high‐affinity clones. After three selection cycles, individual colonies were randomly picked and screened by phage enzyme‐linked immunosorbent assay (phage ELISA) to assess their binding specificity toward RABV‐G. Phage clones exhibiting strong antigen‐specific signals relative to negative controls were selected for sequence analysis and further evaluation.

### 2.4. Phage Rescue and Phage ELISA

*E. coli* XL1‐blue cells harboring phagemids were inoculated into 2×YT medium supplemented with 100 μg/mL ampicillin and 2% glucose and cultured at 250 rpm until the optical density (OD) at 600 nm (OD600) reached ~0.7. VCSM13 helper phage (10^9^ pfu/mL) was then added at a multiplicity of infection (MOI) of ~20:1, followed by incubation at 37°C with shaking for at least 12 h. The cultures were centrifuged at 3000 × g for 15 min to remove bacterial cells, and the phage‐containing supernatant was collected. Phages were precipitated by the addition of 4% (w/v) polyethylene glycol (PEG 8000) and 0.5 M NaCl, incubated on ice for 1 h, and pelleted by centrifugation at 3000 × *g* for 15 min. The phage pellets were resuspended in 1% bovine serum albumin (BSA) in Tris‐buffered saline (TBS) containing 0.02% sodium azide and stored at 4°C until use. For phage ELISA, high‐binding 96‐well microtiter plates (Corning, USA) were coated overnight at 4°C with purified RABV‐G (G protein) at a concentration of 2 μg/mL in carbonate‐bicarbonate buffer (pH 9.6), 100 μL per well. Wells were washed three times with PBS containing 0.05% Tween‐20 (PBST) and blocked with 200 μL of 2% BSA in PBS for 2 h at 37°C. Phage suspensions were diluted 1:1 with 6% BSA/PBS, and 100 μL was added to each well, followed by incubation at 37°C for 2 h. After washing three times with PBST, 100 μL of HRP‐conjugated anti‐M13 antibody (GE Healthcare) diluted 1:5000 in 2% BSA/PBS was added and incubated for 1 h at 37°C. Plates were washed three times with PBST, and 100 μL of TMB substrate (Sigma Aldrich) was added to each well. Color development proceeded for 30 min at 37°C in the dark, and the reaction was stopped with 50 μL of 2M H_2_SO_4_. Absorbance was measured at 450 nm using a microplate reader (BioTek, USA). Clones with OD_450_ values at least 2‐fold higher than background were considered positive.

### 2.5. Recombinant IgG Expression and Purification

Based on sequence analysis of scFv clones showing strong reactivity toward the RABV‐G, the VH and VL domains were selected for conversion into full‐length human IgG1 antibodies. The coding sequences were optimized for mammalian expression using GeneOptimizer 9.0 (Thermo Fisher Scientific) to achieve a codon adaptation index (CAI) greater than 0.85. Each construct incorporated a Kozak sequence (GCCACC) upstream of the start codon to enhance translation efficiency. The optimized VH and VL fragments were cloned into pcDNA3.1‐CH and pcDNA3.1‐CL vectors containing the corresponding human IgG1 constant regions. All recombinant plasmids were verified by restriction digestion and sequencing (Sangon Biotech, Shanghai, China).

For antibody expression, Expi293F suspension cells (Thermo Fisher Scientific) were cotransfected with equal amounts of heavy‐ and light‐chain plasmids using the ExpiFectamine 293 transfection kit, following the manufacturer’s recommendations. Cultures were maintained at 37°C with 8% CO_2_ under orbital shaking (125 rpm) for 5–7 days. The culture supernatants were harvested by centrifugation at 3000 × *g* for 15 min, followed by filtration through 0.22 μm membranes. Recombinant IgG1 antibodies were purified from the clarified medium using Protein A affinity chromatography (GE Healthcare). Bound antibodies were eluted with 0.1 M glycine‐HCl buffer (pH 2.7) and immediately neutralized with 1 M Tris‐HCl (pH 8.0). The eluates were buffer‐exchanged into PBS (pH 7.4) and concentrated using 10 kDa molecular weight cutoff centrifugal filters (Millipore) at 4°C. Purified antibodies were quantified by spectrophotometry at 280 nm, aliquoted, and stored at 80°C until further analysis.

### 2.6. Virus Neutralization Assay

Virus neutralization tests were performed using the ERA strain expressing EGFP. Fifty microliters of each sample, including purified mAbs generated in Section 2.5, the positive reference control (0.5 IU/mL), and the negative control from the National Reference Laboratory for Rabies, were added into four consecutive wells of a 96‐well plate and subjected to serial two‐fold dilution. An equal volume (50 μL) of RABV‐ERA‐EGFP containing 200 TCID_50_/50 μL was added to each dilution and incubated for 1 h at 37°C to facilitate the antibody–virus interaction. Following incubation, BHK‐21 cell suspensions were added to each well and maintained under standard culture conditions for 72 h (the assay was performed in accordance with WOAH guidelines with minor laboratory‐specific optimization of the incubation time). Plates were examined under a fluorescence microscope, and the neutralizing antibody titers were calculated in IU/mL by comparison with the reference standard, as described previously [27].

### 2.7. SDS–PAGE and Western Blot Analysis

The integrity and molecular size of purified mAbs were assessed by SDS–PAGE under both reducing and nonreducing conditions. For reducing analyses, antibody samples were pretreated with dithiothreitol (DTT) or β‐mercaptoethanol, whereas nonreducing samples were loaded directly. Proteins were separated using 12% polyacrylamide gels and visualized with Coomassie Brilliant Blue staining. To evaluate antibody binding to native viral proteins, purified RABV‐G virions were also under both reducing and nonreducing conditions and transferred to nitrocellulose membranes. After blocking with 5% skim milk for 1 h at room temperature, the membranes were incubated overnight at 4°C with mAb H81L90 (0.5 μg/mL), positive control using Ormutivimab (NM57, MCE, HY‐P99792), followed by HRP‐conjugated anti‐human IgG (Abcam, #99759) for 1 h. Protein bands were detected by enhanced chemiluminescence.

### 2.8. Surface Plasmon Resonance (SPR) Analysis

Binding kinetics between RABV‐G and mAb H81L90 were determined using a Biacore T200 system (GE Healthcare). RABV‐G was immobilized onto a CM5 sensor chip via standard amine coupling at a final density of ~2000–3000 response units (RU). Serial dilutions of H81L90 were prepared in 1× EP buffer (pH 7.4) and injected over the chip surface at 30 μL/min. Each cycle consisted of a 90s association phase followed by a 120 s dissociation phase. After each injection, bound analytes were removed using 10 mM glycine–HCl (pH 1.5), and the chip surface was re‐equilibrated with the running buffer. Sensorgrams were processed by subtracting reference channel responses, and kinetic constants (ka and kd) were derived by globally fitting the data to a 1:1 Langmuir binding model using Biacore T200 Evaluation Software. The equilibrium dissociation constant (KD) was calculated from these parameters.

### 2.9. Antigenic Site Analysis

A three‐dimensional structural model of the RABV‐G dimer was generated using the SWISS‐MODEL automated homology modeling platform [28]. The molecular structure of H81L90, initially prepared in the MOL2 format, was converted into a PDB file and subjected to energy minimization using the MM2 force field [29]. The optimized antibody model was then transformed into the PDBQT format for docking. Protein–antibody docking simulations were conducted using AutoDock 4.2 with the Lamarckian genetic algorithm, generating 10 predicted binding conformations for each ligand [30]. Resulting complexes were visualized and analyzed in PyMOL to identify binding residues, hydrogen‐bonding interactions, and structural correlations across antigenic sites II, III, and IV.

### 2.10. Statistics Analysis

All statistical analyses were performed using GraphPad Prism Version 10. Differences with *p*  < 0.05 were considered statistically significant, with *p*  < 0.01 denoting high statistical significance. Three‐dimensional structural figures were prepared in PyMOL, and protein–protein interaction parameters were further analyzed using the Biacore T200 software suite.

### 2.11. PEP in Mice

On day 0, 100 3‐week‐old, sex‐matched BALB/c mice were intramuscularly inoculated in the right gastrocnemius muscle with 50 μL of CVS‐11 strain RABV (100 TCID_50_/mL) [31]. This dose was determined based on preliminary titration experiments to achieve consistent lethal infection (100% mortality) in untreated control mice within 10–14 days postinoculation and is consistent with previously reported murine rabies challenge models. Animals were randomly assigned into 10 experimental groups; random assignment was performed using computer‐generated random numbers. Negative control groups (*n* = 10) received PBS under the same conditions. Five groups (*n* = 10 per group) were treated intramuscularly (in the left gastrocnemius) with recombinant mAb H81L90 50 μL (20 IU/kg, equivalent to 1 mg/kg) at different time points: 24 or 12 h before infection, at the time of infection (0 h), or 12 or 24 h postinfection. A positive control group received PBS in the left hind limb at 0 h prior to infection. Three additional groups (*n* = 10 per group) were administered mAb H81L90, at doses of 0.03 mg/kg, 0.06 mg/kg, or 1 mg/kg, respectively, at 0 h postinfection. All animals were monitored twice daily for signs of rabies, including motor impairment, ataxia, paralysis, or decreased responsiveness. Mice exhibiting any of these symptoms were humanely euthanized. Brain tissues were collected and examined using the fluorescent antibody test (FAT), involving impression smears fixed in acetone, stained with mouse mAb [RV1C5] (Catalog: ARG54569, Arigo), Goat anti‐mouse IgG (H + L) Highly Cross‐Adsorbed Secondary Antibody, Alexa Fluor Plus 488‐A32723 (Catalog: A32723, Invitrogen), and observed under fluorescence microscopy as per WOAH standards [32].

## 3. Results

### 3.1. Construction of a Fully Human Anti RABV‐G Phage Display Library

**Figure 1:**
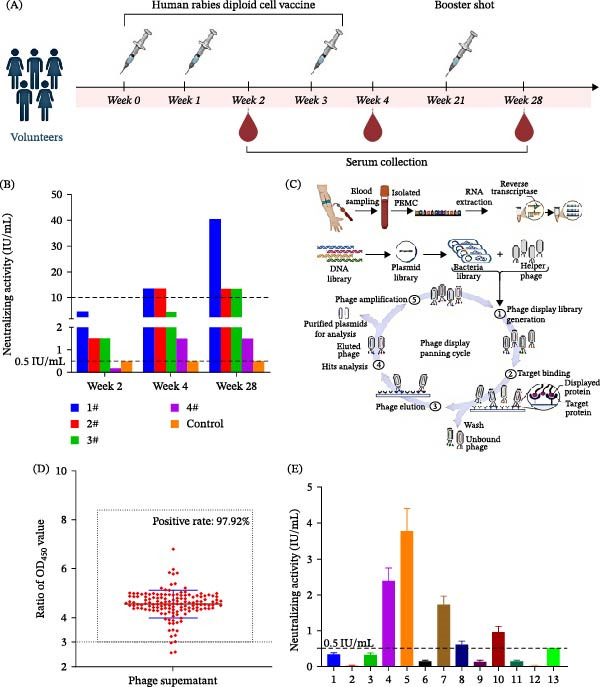
Construction and screening of fully human anti‐RABV‐G phage display library. (A) Schematic representation of the workflow for generating the immune antibody library from human donors immunized with rabies vaccine. (B) Neutralizing antibody titers in serum samples collected after each vaccination. Three donors with titers exceeding 10 IU/mL were selected for library construction. (C) Schematic diagram of the constructed phage display antibody library, comprising both λ and κ light chain libraries. The capacities of the scFv‐λ and scFv‐κ sublibraries were 5.1 × 10^7^ pfu/mL and 7 × 10^6^ pfu/mL, respectively. (D) Screening results of 144 randomly selected clones by phage ELISA, demonstrating a positive rate of 97.9%. (E) Identification of candidate clones with neutralizing activity by fluorescent antibody virus neutralization test (FAVN), highlighting five clones with titers >0.5 IU/mL, including two clones exceeding 2 IU/mL.

To generate fully human mAbs capable of neutralizing RABV, four volunteers who had received at least three doses of a commercially available rabies vaccine were recruited. Neutralizing antibody titers were monitored after each immunization (Figure 1A), and three individuals with titers exceeding 10 IU/mL (Figure 1B) were selected for library construction. From each selected donor, 100 mL of anticoagulated blood was collected, and PBMCs were isolated for total RNA extraction and reverse transcription. The resulting cDNA was then used as the template for phage‐display library construction (Figure 1C). The scFv‐λ and scFv‐κ libraries exhibited capacities of 5.1 × 10^7^ pfu/mL and 7 × 10^6^ pfu/mL, respectively, with an overall positive clone rate of 95%.

### 3.2. Identification of Fully Human Anti‐RABV‐G scFvs

To identify neutralizing antibodies (Nbs) specific to the RABV‐G, 144 monoclonal phage supernatants were randomly selected and screened by phage ELISA. Samples were considered positive when the OD_450_ ratio of the sample to the negative control exceeded 3. A high positive rate of 97.92% (141/144) was observed (Figure 1D). Phage supernatants with OD_450_ ratios greater than five were further processed for endotoxin removal and subsequently evaluated for neutralizing activity by FAVN. Five candidates with neutralizing titers exceeding 0.5 IU/mL were observed, among which two clones exhibited titers greater than 2 IU/mL (Figure 1E).

### 3.3. Expression and Characterization of Recombinant mAbs

**Figure 2:**
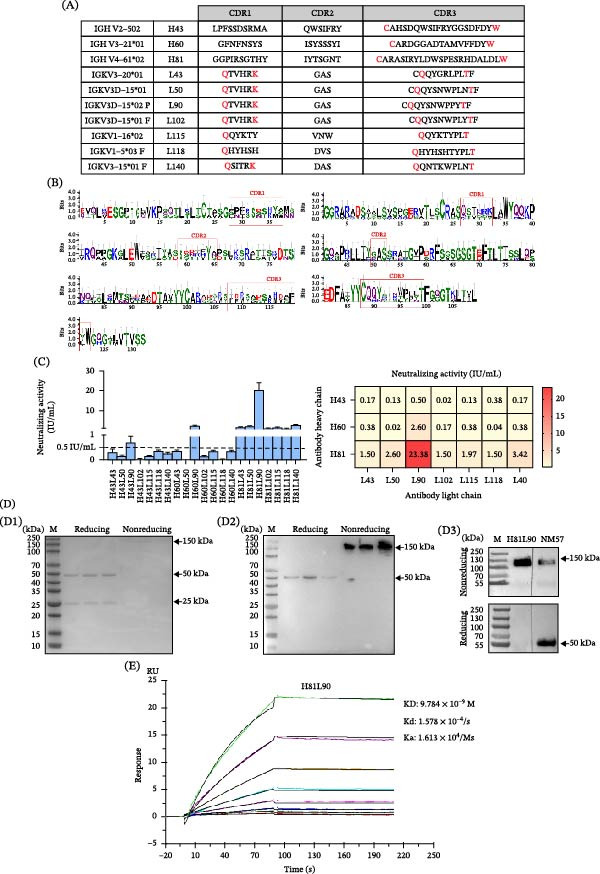
Sequence analysis and functional evaluation of recombinant monoclonal antibodies. (A) IMGT/V‐QUEST analysis of variable regions revealed three distinct VH and seven VL sequences, all belonging to the κ light chain family. (B) Repertoire analysis indicated conserved residues at key CDR positions (e.g., H‐CDR3 C100 and W122, L‐CDR1 Q27 and K33, L‐CDR3 Q89 and T99), suggesting preferential mutation patterns during affinity maturation. (C) Functional reconstitution of 21 candidate antibodies in HEK‐293F cells, with nine displaying measurable neutralizing activity, among which H81L90 (VH‐B81/VL‐B90) exhibited the highest potency (20 IU/mL). (D) SDS–PAGE analysis of purified H81L90 under nonreducing and reducing conditions (D1), Western blot analysis of purified H81L90 under nonreducing and reducing conditions (D2), and Western blot analysis of recombinant RABV‐G probed with H81L90 and the positive control antibody NM57 under nonreducing and reducing conditions (D3). H81L90 recognized recombinant RABV‐G under nonreducing conditions but not under reducing conditions, whereas NM57 detected recombinant RABV‐G under both conditions. (E) SPR analysis demonstrated nanomolar affinity of H81L90 for RABV‐G, with KD = 9.784 × 10^−9^ M.

Selected scFvs with an OD450 ratio exceeding 5 between the sample and negative control (see Figure 1D), which were used in the IMGT/V‐QUEST database, revealed three unique VH‐chain and seven VL‐chain sequences, all of which belonged to the κ‐chain family (Figure 2A). Germline gene analysis showed that the VH sequences originated from distinct lineages, whereas most VL sequences were clustered within a single lineage, suggesting a broad yet structured diversity of antibody repertoires within the constructed library. Affinity maturation during antibody evolution is typically associated with the gradual accumulation of somatic mutations, a process tightly correlated with enhanced binding strength and neutralizing capability [33]. The average lengths of the complementarity‐determining regions (CDRs) in VH were 9.3 (CDR1), 7.3 (CDR2), and 18.0 (CDR3) amino acids, while those in VL measured 6.0, 3.0, and 10.7 amino acids, respectively. Detailed repertoire profiling showed that certain residues within the germline‐derived CDRs displayed both mutational hotspots and high conservation. The Weblogo alignment was generated using the three unique VH and seven VL sequences identified from the selected scFvs via IMGT/V‐QUEST. Notably, residues Cys100 and Trp122 in H‐CDR3, Gln27 and Lys33 in L‐CDR1, as well as Gln89 and Thr99 in L‐CDR3, frequently appeared among high‐frequency variants (Figure 2B). The conservation of these residues suggests a structural role in maintaining loop integrity and antigen binding, whereas variability at neighboring sites may facilitate affinity maturation and fine‐tuning of antigen specificity. Neutralization was considered measurable when titers were ≥0.5 IU/mL in FAVN. Based on the 3 × 7 heavy/light reconstitutions (*n* = 21), nine combinations met this criterion (Figure 2C). Among these, H81L90, formed by pairing heavy‐chain B81 with light‐chain B90, exhibited the strongest neutralizing potency, with a titer of 20 IU/mL. Remarkably, all antibodies incorporating the B81 heavy chain displayed enhanced neutralization, highlighting the dominant contribution of this VH region to functional efficacy. To further verify the antibody structure, we conducted an analysis of the expression of H81L90 through SDS–PAGE and evaluated its specific binding to the RABV‐G protein by means of Western blot (Figure 2D). Under nonreducing conditions, the H81L90 antibody displays a band at ~150 kDa, which is consistent with the intact IgG. Under reducing conditions, it distinctly dissociates into a heavy chain of around 50 kDa and a light chain of ~25 kDa, validating its proper assembly and the integrity of disulfide bonds. These findings are highly congruent with the anticipated molecular weight, corroborating the reliability of its structural and functional characteristics (D1,D2). The Western blot results used to evaluate the epitope‐recognition property of H81L90 toward recombinant RABV‐G. Under nonreducing conditions, H81L90 specifically recognized recombinant RABV‐G at ~150 kDa, with a clear signal and a low background. In contrast, under reducing conditions, no detectable RABV‐G band was observed with H81L90. As a positive control, NM57 recognized recombinant RABV‐G under both nonreducing and reducing conditions(D3). These results confirm the specific binding reactivity of H81L90 toward recombinant RABV‐G and further demonstrate that the epitope recognized by H81L90 depends on the preservation of disulfide bond‐mediated structural integrity, supporting its classification as a conformational epitope rather than a linear epitope.

### 3.4. Interaction Analysis of H81L90 With RABV‐G by SPR

The binding kinetics between RABV‐G and the fully human mAb H81L90 were quantified using SPR. The G‐protein was immobilized on a CM5 sensor chip via amine coupling with an immobilization level of 788 RU. Serial dilutions of H81L90 (1000–7.81 nM) were injected at a flow rate of 30 μL/min with 90s association and 120 s dissociation phases. Global fitting of the data to a 1:1 Langmuir model yielded an association rate constant (Ka) of 1.613 × 10^4^/M/s, a dissociation rate constant (Kd) of 1.578 × 10^−4^/s, and an equilibrium dissociation constant (KD) of 9.784 × 10^−9^ M, indicating strong nanomolar affinity (Figure 2E).

### 3.5. Epitope Recognition Analysis of mAb H81L90 on RABV‐G

**Figure 3:**
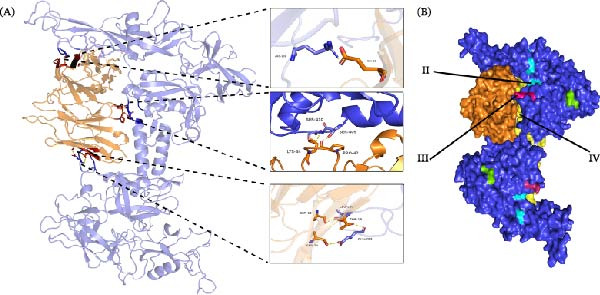
Epitope mapping and binding kinetics of monoclonal antibody H81L90. (A) Molecular docking analysis identified 5 RABV‐G residues (Arg103, Leu290, Glu293, Ser409, and Ser410) as key hydrogen‐bonding sites. (B) Structural mapping of antigenic sites II, III, and IV reveals that H81L90 engages multiple epitope clusters, reducing the likelihood of viral escape.

To further characterize the epitope specificity of H81L90, molecular docking and hydrogen‐bond analyses were performed to elucidate its interaction with the RABV‐G (Figure 3A). Five amino acid residues—Arg103 (heavy‐chain‐mediated), Leu290 and Glu293 (heavy‐chain‐mediated), Ser409, and Ser410 (light‐chain‐mediated)—were identified as key hydrogen‐bonding sites mediating H81L90 binding. Structural mapping revealed that these residues are distributed across multiple antigenic regions of RABV‐G, including sites II, III, and IV (Figure 3B). The heavy chain primarily drives interactions with site II (e.g., Arg103) and site IV (e.g., Leu290 and Glu293), aligning with its substantial contribution to neutralization as evidenced in Figure 2C, while the light chain mediates supportive contacts near site III (e.g., Ser409 and Ser410). Within site II, Arg103 was located in the structural scaffold. Engagement of H81L90 with these residues is therefore likely to impede viral receptor binding, thereby enhancing its neutralizing potency. Importantly, these residues cluster into a single continuous conformational surface on prefusion RABV‐G that spatially overlaps regions proximal to antigenic sites II/III/IV rather than representing multiple independent epitopes. Collectively, these findings demonstrate that H81L90 has been highlighting its potential as a protective neutralizing antibody.

### 3.6. mAb H81L90 Protects Mice From Lethal RABV Infection

**Figure 4:**
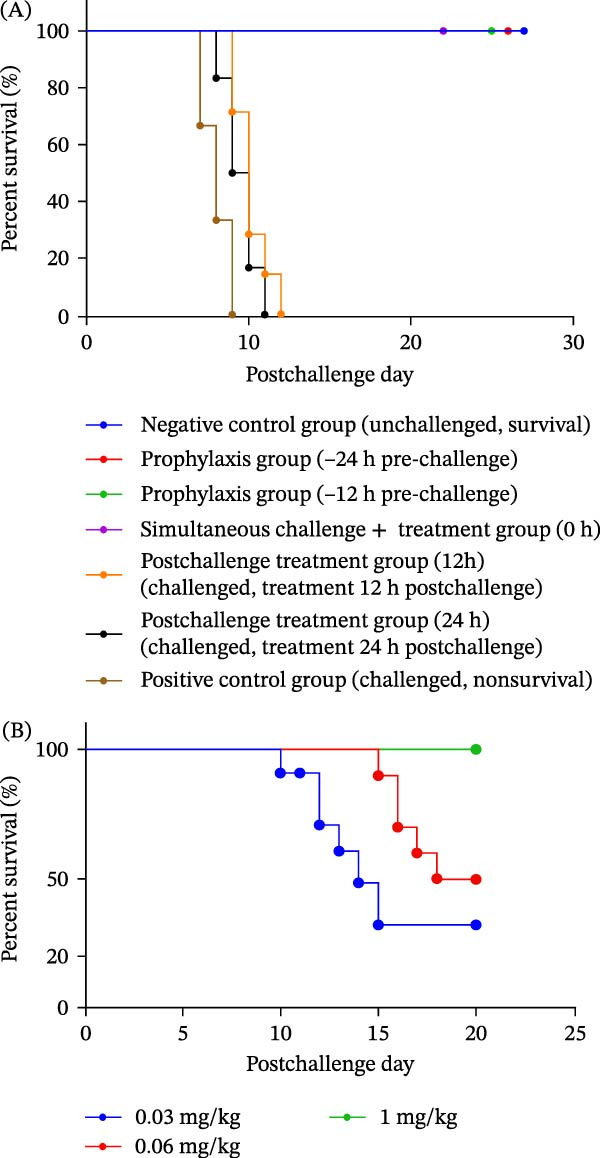
Protective efficacy of monoclonal antibody H81L90 against lethal RABV challenge in mice. (A) Survival curves of BABL/C mice administered with H81L90 for prophylaxis or postexposure treatment following intramuscular inoculation with a lethal dose of CVS‐11 strain. All animals in the negative control group (unchallenged, PBS‐treated) survived, whereas 100% of the positive control mice (challenged, nontreated) succumbed between 11 and 14 days postchallenge. Complete protection (100% survival) was observed when H81L90 was administered 24 or 12 h before, or simultaneously (0 h) with viral challenge. In contrast, delayed administration at 12 or 24 h postinfection failed to protect the animals. (B) Dose‐dependent postexposure protection by H81L90. At 1 mg/kg, all mice (10/10) survived; administration of 0.06 mg/kg resulted in 50% survival (5/10), and 0.03 mg/kg protected 30% (3/10) of challenged mice, demonstrating that H81L90 retained neutralizing activity even at low antibody concentrations.

To evaluate the in vivo neutralizing activity of the mAb H81L90, BABL/C mice were employed to assess both pre‐ and postexposure protection against lethal RABV infection. Groups of 10 mice were intramuscularly inoculated in the hind limb with a lethal dose of CVS‐11 virus, followed by administration of H81L90 for prophylactic or postexposure prophylactic assessment. All mice in the positive control group (challenged, nonsurvival) (100%) and those receiving H81L90 at 12 or 24 h postinfection (100%) succumbed between days 11 and 14. In contrast, mice treated with H81L90 at −24, −12, or 0 h relative to infection showed complete protection, with 100% survival throughout the 25‐day observation period. Postexposure protection of H81L90 was time‐dependent (Figure 4A). At 1 mg/kg, H81L90 conferred full protection (10/10, 100%), while at 0.06 mg/kg, it resulted in 50% (5/10) survival, and even at the lowest dose (0.03 mg/kg), 30% of mice (3/10) survived. These findings indicate that H81L90 retains partial protective activity even at low concentrations (Figure 4B); although the antibody concentration was below the threshold required for complete protection, it was sufficient to partially limit viral spread, indicating a dose‐dependent protective effect, consistent with previous findings that mAbs can provide effective PEP against RABV [34].

**Figure 5:**
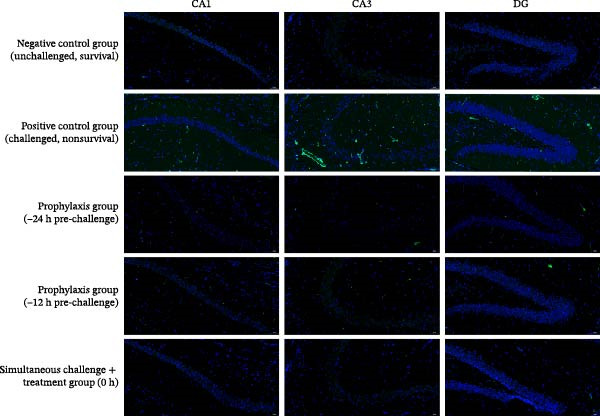
Representative immunofluorescence images of rabies viral antigen in mouse hippocampus. Nuclei were counterstained with DAPI (blue). Strong, characteristic neuronal cytoplasmic fluorescence was observed in the positive‐control group. In contrast, only weak, sporadic background‐level signals without typical neuronal distribution were observed in mice receiving prophylactic or simultaneous H81L90 treatment. The marked reduction in viral signal indicates efficient viral clearance within the central nervous system.

To further assess viral clearance in the central nervous system, brain tissues from surviving mice and PBS controls were examined by immunofluorescence. In the hippocampal CA1, CA3, and dentate gyrus (DG) regions, green fluorescence signals were markedly reduced in H81L90‐treated groups compared with positive controls. The clear distinction between experimentally treated and control animals demonstrates efficient viral neutralization at infection sites. Although minor regional variations were observed in the proportion and density of positive cells, the mean OD values remained consistent across replicates, supporting the reproducibility and reliability of the H81L90 antiviral effect in hippocampal immunofluorescence assays (Figure 5). Rabies viral antigen was readily detectable in the hippocampi of positive‐control mice, showing characteristic granular cytoplasmic fluorescence within neurons. In contrast, only sporadic, weak fluorescent signals at the background level were observed in animals receiving prophylactic or simultaneous H81L90 treatment, and no typical rabies antigen‐positive neuronal structures were identified. These results demonstrate that H81L90 provides potent prophylactic and postexposure prophylactic protection against lethal rabies infection in mice, underscoring its potential as an effective alternative or complement to conventional RIGs and vaccines.

## 4. Discussion

The present study identifies H81L90 as a fully human mAb with promising potential for activity against RABV, offering a promising alternative to conventional RIG for PEP. Our findings highlight the feasibility of recombinant antibody technologies to overcome the limitations of plasma‐derived RIG products, particularly in regions where limited availability, variable potency, and high cost restrict effective rabies prevention. By targeting the viral glycoprotein (RABV‐G), H81L90 effectively neutralized virus entry both in vitro and in vivo, providing complete protection in a lethal murine challenge model. H81L90 displayed high binding affinity for RABV‐G, with kinetic parameters in the nanomolar range, consistent with those of previously described broadly neutralizing antibodies, such as RVC20 and RVC58 [10, 24]. Structural analysis revealed that H81L90 recognizes a conformational epitope, and contact residues are located in proximity to regions assigned to antigenic sites II, III, and IV. These regions include residues critical for receptor binding and fusogenic activation, suggesting that H81L90 interferes with structural rearrangements necessary for membrane fusion. Site III, which harbors the neurovirulence‐associated residue Arg333, is particularly significant as antibodies targeting this region often exhibit broad neutralization across diverse RABV lineages [8, 9]. The ability of H81L90 to recognize a conformational epitope spanning multiple antigenic sites likely restricts the emergence of escape mutants, a common challenge in single‐epitope‐targeting antibodies. While this study provides a predictive model of the H81L90/RABV‐G complex based on computational docking, such in silico epitope assignment remains hypothesis‐generating. The precise structural epitope and interface will require experimental validation using structural biology approaches (cryo‐electron microscopy or X‐ray crystallography), complemented by functional assays such as competition binding and escape mutant mapping.

In vivo, H81L90 provided robust protection when administered before or at the time of viral exposure, consistent with its strong neutralizing capacity observed in vitro and with previous findings that pre‐ or postexposure administration of mAbs can provide effective prophylaxis against RABV [33]. Notably, partial protection was retained even when treatment was delayed, indicating that H81L90 can mitigate disease progression after the initial infection. This property parallels the dose‐dependent protective effects reported for clinically advanced mAbs such as SYN023 and Ormutivimab in preclinical and clinical studies [16, 19]. While post‐12/24 h treatments failed, H81L90 treatment vaccination significantly prolonged the survival duration in mice compared to the positive control group. Once RABV enters peripheral neurons and initiates retrograde axonal transport, extracellular neutralizing antibodies have limited access to intracellular virus. This indicates that H81L90 exhibits the capacity to delay disease progression even in scenarios involving partial viral establishment, thereby offering experimental support for expanding the therapeutic time window for PEP. It should be noted that the current in vivo design evaluated H81L90 as a standalone intervention to define its intrinsic neutralizing and protective capacities. Therefore, the protective window observed here reflects the intrinsic capacity of H81L90 to neutralize virus prior to neuronal entry, rather than its ability to bridge vaccine‐induced immunity. This approach does not fully replicate the standard clinical PEP regimen, which combines vaccination with passive immunization. Future studies will assess the efficacy of H81L90 in combination with rabies vaccines under clinically relevant exposure‐to‐treatment intervals. Such characteristics are especially relevant for real‐world scenarios, where access to PEP may be delayed and HRIG remains scarce and costly in many endemic regions [35]. Histopathological analysis confirmed markedly reduced viral antigen accumulation in the hippocampal neurons of treated mice, implying that the antibody effectively limits neuroinvasion, a crucial step in rabies pathogenesis. These findings align with previous reports demonstrating that antibodies capable of intercepting RABV at peripheral sites can prevent subsequent CNS dissemination [7].

Beyond its biological performance, H81L90 possesses several practical advantages that make it suitable for large‐scale clinical applications. As a fully human IgG1, it minimizes the risk of hypersensitivity and serum sickness associated with heterologous proteins, a frequent complication of ERIG. Its recombinant nature ensures uniform potency, eliminates dependance on plasma donors, and facilitates scalable manufacturing under good manufacturing practice (GMP) conditions. These characteristics are critical for ensuring a consistent global supply, an enduring challenge that has limited HRIG distribution for decades [35]. Furthermore, as production technologies for mAbs continue to advance, manufacturing costs are expected to decline, enhancing accessibility in resource‐limited settings.

The development of antibody‐based biologics for rabies PEP has progressed considerably in recent years. Licensed products such as Rabishield (17C7) and Twinrab (a cocktail of two distinct mAbs, a cocktail antibody) have demonstrated that mAbs can effectively replace RIG in human PEP regimens [15, 16]. However, the antigenic diversity of circulating RABV strains underscores the importance of identifying antibodies with broader cross‐lineage reactivity. H81L90’s epitope profile, which includes conserved residues involved in receptor engagement, suggests potential for broad‐spectrum neutralization; nevertheless, further validation against diverse field isolates representing different phylogenetic lineages is warranted to fully assess its breadth of coverage [18]. A limitation of this study is the absence of a direct HRIG comparator group. Although HRIG efficacy has been well established in similar murine models, future studies will include head‐to‐head comparisons to further assess the translational potential of H81L90.

Another key factor for clinical translation is the antibody’s pharmacokinetic and biodistribution profiles. For optimal PEP efficacy, antibodies must reach the inoculation site rapidly to neutralize virus particles before neuronal entry. Future studies should evaluate H81L90’s tissue penetration and half‐life following intramuscular administration in larger animal models, as well as define its pharmacodynamic properties and safety margin [18]. In addition, combination therapy represents a rational strategy to enhance protection. The World Health Organization recommends antibody cocktails targeting nonoverlapping epitopes to reduce the risk of viral escape and broaden lineage coverage [12]. Additionally, structural studies, such as cryo‐electron microscopy, will be invaluable for elucidating the precise molecular interactions between H81L90 and RABV‐G. Understanding whether the antibody stabilizes the prefusion state of the glycoprotein—similar to RVC20 and RVC58—could provide insights into its mechanism of action and guide the design of complementary antibodies for cocktail formulations [10].

In conclusion, H81L90 demonstrates high affinity and potent in vivo protection, establishing it as a promising candidate for next‐generation rabies PEP. Its recombinant nature, favorable safety profile, and scalability position it as a viable alternative to HRIG and ERIG. The continued development and clinical translation of such antibodies represent a major step toward improving access to effective rabies prophylaxis and advancing the WHO’s 2030 goal for the global elimination of human rabies [20].

## Author Contributions

**Ruo Mo**: writing – original draft, visualization, validation, methodology, investigation, conceptualization. **Jingqi Xu, Huanqin Zheng, and Tianyi Yin**: validation, methodology, investigation. **Jianzhong Wang, Na Feng, Tiecheng Wang, Feihu Yan, Haiyang Cong, and Lugong Chen:** validation, supervision, methodology, investigation. **Yongkun Zhao and Xianzhu Xia:** review and editing, supervision, methodology, conceptualization, foundation.

## Funding

This study was supported by the Jilin Provincial Scientific and Technological Development Program (Grant 20210202052NC).

## Disclosure

All authors reviewed and edited the manuscript and have read and agreed to the funding statement.

## Ethics Statement

This study was approved by the Ethics Committee of the Changchun Veterinary Research Institute, Chinese Academy of Agricultural Sciences (Approval Number AF/SC‐08/02.448). All participants provided written informed consent prior to sample collection. The study was conducted in accordance with the Declaration of Helsinki. All animal experiments were approved by the Animal Welfare and Ethics Committee of the Changchun Veterinary Research Institute, Chinese Academy of Agricultural Sciences, and the College of Veterinary Medicine, Jilin Agricultural University (Approval Number IACUC/JSYAL‐11‐2025‐056). All procedures were conducted in accordance with the Guidelines for Ethical Review of Animal Welfare (GB/T 35892‐2018) and complied with the standards of the Association for Assessment and Accreditation of Laboratory Animal Care (AAALAC). Experimental designs were carefully planned to minimize animal discomfort, pain, and distress whenever possible.

## Conflicts of Interest

The authors declare no conflicts of interest.

## Data Availability

The data that support the findings of this study are available from the corresponding author upon reasonable request.
